# Tumor Ablation with Irreversible Electroporation

**DOI:** 10.1371/journal.pone.0001135

**Published:** 2007-11-07

**Authors:** Bassim Al-Sakere, Franck André, Claire Bernat, Elisabeth Connault, Paule Opolon, Rafael V. Davalos, Boris Rubinsky, Lluis M. Mir

**Affiliations:** 1 CNRS UMR 8121, Institut Gustave-Roussy, Villejuif, France; 2 University Paris-Sud, UMR 8121, Villejuif, France; 3 School of Biomedical Engineering and Sciences, Virginia Tech-Wake Forest University, Blacksburg, Virginia, United States of America; 4 Department of Bioengineering, University of California at Berkeley, Berkeley, California, United States of America; 5 Department of Mechanical Engineering and Graduate Program in Biophysics, University of California at Berkeley, Berkeley, California, United States of America; 6 Center for Bioengineering in the Service of Humanity and Society, School of Computer Science and Engineering, Hebrew University of Jerusalem, Givat Ram, Jerusalem, Israel; Center for Genomic Regulation, Spain

## Abstract

We report the first successful use of irreversible electroporation for the minimally invasive treatment of aggressive cutaneous tumors implanted in mice. Irreversible electroporation is a newly developed non-thermal tissue ablation technique in which certain short duration electrical fields are used to permanently permeabilize the cell membrane, presumably through the formation of nanoscale defects in the cell membrane. Mathematical models of the electrical and thermal fields that develop during the application of the pulses were used to design an efficient treatment protocol with minimal heating of the tissue. Tumor regression was confirmed by histological studies which also revealed that it occurred as a direct result of irreversible cell membrane permeabilization. Parametric studies show that the successful outcome of the procedure is related to the applied electric field strength, the total pulse duration as well as the temporal mode of delivery of the pulses. Our best results were obtained using plate electrodes to deliver across the tumor 80 pulses of 100 µs at 0.3 Hz with an electrical field magnitude of 2500 V/cm. These conditions induced complete regression in 12 out of 13 treated tumors, (92%), in the absence of tissue heating. Irreversible electroporation is thus a new effective modality for non-thermal tumor ablation.

## Introduction

Minimally invasive tissue ablation has become of central importance in the modern surgery armamentarium. In the treatment of benign or malignant tumors it is important to achieve ablation of the undesirable tissue in a well-controlled and precise way without affecting the surrounding healthy tissue. As an alternative to surgical resection, a number of minimally invasive methods have been developed to destroy specific areas of undesirable tissues. Most of these techniques are thermal using cold, e.g. cryosurgery [1]–[3] or heat, e.g. radiofrequency [4], [5].

Electroporation, also known as electropermeabilization, is a term used to describe the permeabilization of the cell membrane as a consequence of the application of certain short and intense electric fields across the cell membrane, the cells or the tissues. The permeabilization can be temporary (reversible electroporation) or permanent (irreversible electroporation) as a function of the electrical field magnitude and duration, and the number of pulses [6]. Reversible electroporation is commonly used *in vitro* to facilitate the penetration of various otherwise non-permeable macromolecules across the cell membrane [7]–[9]. Irreversible electroporation, the ability of certain electrical pulses to permanently permeabilize the cell membrane, has been known for over three decades. For most of this period irreversible electroporation (IRE) was used primarily for ablation of microorganisms and cells *in vitro* and studied only as an upper limit of electrical parameters for reversible tissue electroporation applications. Our group has pursued the understanding of the electrical fields and processes that produce IRE with single cell micro-electroporation technology [10], [11].

The study of Davalos, Mir and Rubinsky, which showed that IRE can ablate substantial volumes of tissue without inducing a thermal effect and therefore serve as an independent and new tissue ablation modality, opened the way to the use of IRE in surgery [12]. Subsequently, Edd *et al.* demonstrated tissue ablation with IRE *in vivo* in the normal liver of rats. [13]. Complete ablation of the targeted liver tissue was achieved by exposing the tissue to electrical parameters that do not induce thermal damage [13]. Massive blood vessel congestion was observed in the sinusoids of the treated volume, which should significantly enhance the treatment. The study concluded that IRE produces precisely delineated ablation zones with cell scale resolution between ablated and non-ablated areas and the ability of mathematical modelling to precisely predict the ablated area. A more recent study was performed to evaluate the long term effects of IRE in a large animal model [14]. The results demonstrated the ability of electroporation to ablate large volumes of tissue using electrical parameters that while substantially above those conventionally used in reversible electroporation do not induce substantial thermal effects. The histology has reconfirmed the results in Davalos *et al.* [12] and Edd *et al.* [13] showing that mathematical modeling of electrical and thermal fields are a powerful tool in designing IRE ablation protocols, that IRE can be used to ablate tissue with cell scale resolution and that indeed IRE affects only the cell membrane and therefore spares connective tissue. Another important finding is that IRE can ablate tissue to the margin of large blood vessels and, unlike thermal ablation, is not affected by blood flow in these vessels. This implies that IRE could become an important modality for treatment of tumors near blood vessels.

IRE is a member of a family of non-thermal methods to ablate tissue with electrical pulses, which includes electrochemotherapy (ECT) [7], [15]–[18] and supra-poration [19]–[21]. ECT is a relatively new minimally invasive tissue ablation technique that employs reversible electroporation pulses (typically a sequence of eight 100 µs pulses of approximately 1000 V/cm) to reversibly permeabilize the cell membrane and thereby facilitate the penetration into cells of small amounts of non-permeant or low-permeant anti-cancer drugs, such as bleomycin or cisplatin. A major advantage of ECT is that the technique selectively kills, through the use of bleomycin, only the dividing tumour cells and spares the normal non-dividing cells. However, by its nature, ECT requires of the use of chemical agents, which IRE does not. In tissue ablation, ECT is proven to be a safe and highly efficient method to introduce non-permeable cancer drugs into malignant cells and is currently used to treat cutaneous and subcutaneous tumors in humans [15], [16], [22]–[24]. The standard operating procedures [17] have been established after a clinical multicenter study [18] and several previous single center clinical trials, as reviewed by G. Sersa [25].

Supra-poration, another non-thermal method to kill tissue, is achieved by means of nanosecond electrical pulses in the tens of nanoseconds range and 40–80 kV/cm of field strength [19], [20]. R. Nuccitelli *et al.* [26] described antitumor effects in mice when nanosecond pulses were delivered in two sets consisting of three consecutive days of treatment separated by two to three weeks. In supra-poration, which also does not employ chemical agents, the pulses delivered are much shorter and the magnitude of the field is higher by an order of magnitude than in IRE. In supra-poration cell death is not a consequence of the irreversible cell membrane permeabilization as in IRE, but the probable result of Ca^2+^ ions released inside the cells from internal Ca^2+^ storage vesicles permeabilized by the nanosecond pulses [20]. Each of these new techniques based on the non thermal delivery of electric pulses, namely ECT, IRE and supra-poration, has inherent advantages and disadvantages for tissue ablation and it is quite likely that each will find appropriate uses in modern medicine, separately or in combination.

The present study is the first report of an attempt to evaluate the effectiveness of IRE in treatment of tumors *in vivo* using preclinical mouse models. Our goal was to determine whether IRE alone, with minimal thermal effects, could actually produce substantial tumor ablation. We also analyzed the cell death and the changes in the vasculature in the treated tissue. The results presented in this study provide further evidence that IRE may become an important minimally invasive modality for treatment of cancer.

## Methods

### Tumour cells culture and tumour production

Cells from a LPB cell line, a methylcholanthrene-induced C57Bl/6 mouse sarcoma cell line [27], were cultured using standard procedures in MEM (Gibco BRL, Cer-gy-Pontoise, France) supplemented with 100 U.ml^−1^ penicillin, 100 mg.ml^−1^ streptomycin (Sarbach, France) and 8% foetal calf serum (Gibco). C57Bl/6 female mice, 6–8 weeks old, were inoculated subcutaneously in the left flank with 1×10^6^ cells, producing in 9 days tumors of 4 to 5mm in diameter. Animals were housed at IGR, handled according to the recommended guidelines [28] and protocols approved at IGR.

### Tumour treatment

At the start of the procedure mice were anaesthetised using a mixture of xylazine 12.5 mg.kg^−1^ (Bayer Pharma, Puteaux, France) and ketamine 125 mg.kg^−1^ (Parke Davis, Courbevoie, France). An incision was performed on the skin near the tumor and the skin flap containing the tumor was lifted, taking particular care to avoid cutting the main blood vessels nourishing the tumor. Stainless-steel plate electrodes were placed in direct contact with both sides of the cutaneous tumor, with the tumor sandwiched between the parallel plates. Good contact of the electrodes with the tumor tissue was produced using electrocardiography paste (Eko-gel, Camina, Egna, Italy). The distance between the electrodes ranged from 3 to 5 mm and was adjusted to tumour size. The spacing between the electrodes was measured and the information was used to set the voltage delivered by the pulse generator, so as to produce the design field magnitude across the tumor, planned for the experiment. The electrical parameters used in the various experiments are listed in Table 1. The square-wave electric pulses (EP) were generated by an electroporation power supply, a Cliniporator™ (Igea, Carpi, Italy) or, to deliver pulses with a frequency of 0.3 Hz, a GHT 1287 (Jouan, St Herblain, France ). To obtain a pulse application frequency of 0.03 Hz, pulses were manually delivered by the operator every 33 s. After EP delivery, the skin incisions were closed with metallic clips, the mice were returned to their cages and the evolution of the treated tumors was followed with measurements of tumor size every second day. Alternatively, mice were kept for different periods of time (between 1 and 72 h) and then humanely sacrificed by CO_2_ inhalation before the tumors were removed and processed for histological or immunohistochemical analysis.

**Table 1:** Details of the experimental conditions tested and summary of the antitumor effects achieved.

Exp	N	t [µs]	U/d [V/cm]	d [mm]	f [Hz]	T_max_ [°C]	t_43_ [s]	CR
α: IMMUNOCOMPETENT MICE (Figure 1)								
1B	1	800	2000	3 or 5	1	39	0.01	0/7 (0%)
1C	8	100	2000	3 or 5	1	38	0.002	0/7 (0%)
1E	8 (4×2)	800	2000	5	1	43	0.6	0/6 (0%)
1F	64 (16×4)	100	2000	5	5000	40	0.03	1/6 (17%)
1H	8 (4×2)	800	2500	4	1	46	30	3/6 (50%)
1I	64( 16×4)	100	2500	4	5000	41	0.3	2/6 (33%)
1K	64(16×4)	100	2500	4	1	38	0.004	3/8 (37%)
1L	8 (4×2)	1000	2500	4	0.3	51	1300	5/8 (62%)
β: IMMUNODEFICIENT MICE (Figure 2)								
2B	8 (4×2)	1000	2500	4	0.3	51	1300	2/13 (15%)
2C	80 (4×20)	100	2500	4	3	40	0.1	11/16 (69%)
2E	8 (4×2)	1000	2500	4	0.03	40	0.05	4/13 (31%)
2F	80 (4×20)	100	2500	4	0.3	37.5	0.001	12/13 (92%)

In all experiments except 1B and 1C, the pulses were delivered in 2 perpendicular orientations. The EP were delivered using a Cliniporator in all conditions except 1L, 2B and 2F for which a Jouan device was used (see Methods). Exp: experimental condition; n: number of pulses; t: individual pulse duration; U/d: voltage to distance ratio (approximate electric field strength); d: distance between the plate electrodes; f: repetition frequency; T_max_: theoretical maximum temperature; t_43_: theoretical equivalent thermal dose; CR: number of complete regressions/number of treated tumors, and percentage of CR.

### Numerical model of temperature distribution and thermal dose assessment

Electrical fields produce thermal effects due to Joule type electrical energy dissipation. Thermal damage occurs when tissue is exposed to a temperature higher than the physiological temperature for an extended period of time. In electroporation the tissue temperature fluctuates during the application of the pulses and between the pulses. For situations involving fluctuations of temperature, an analytical procedure was developed in which thermal damage is assessed by treating the tissue as if it was at a constant temperature, typically 43°C, for a duration that would result in an equivalent thermal effect to the fluctuating temperature [29]. The following expression is used to calculate the duration necessary to hold the tissue at 43°C to result in an equivalent thermal dose to a fluctuating temperature:
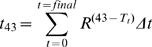
(1)where *T_t_* is the average temperature during Δ*t* with *R* = 0.25 when *T_t_*≤43°C and *R* = 0.5 when *T_t_*>43°C [30], [31] In order to calculate the equivalent thermal dose for each experimental condi-tion, the temperature within the tissue as a function of time was estimated from the Pennes bioheat transfer equation [29] with the addition of a Joule heating term as described in detail in [32].

A one-dimensional explicit transient finite difference model was created to estimate the heating associated with each electroporation procedure [33]. The model geometry was representative of the experimental conditions of a tumour in contact with electrodes, in which the distance between the parallel electrodes was typically 4 mm. The problem was solved under the assumption that the electroporation electrode side facing away from the tumor is cooled by convection heat transfer with the surrounding air and is treated as an infinite fin as described in [32]. The free convection heat transfer coefficient was taken as 15 W m^−^^2^K^−1^, and the ambient air, as 25°C [34]. It is assumed that the tissue and the electrode are initially at 37°C and 25°C, respectively.

The values for the tissue heat capacity (4 kJ kg^−1^K^−1^), electrical conductivity (0.2 S m^−1^), thermal conductivity (0.5 W m^−1^K^−1^) and density (1000 kg m^−3^) are taken from the literature for mouse fibrosarcoma tissue 7 days after inoculation of the tumor cells [35], [36]. The values for the electrode heat capacity (477 J kg^−1^K^−1^), electrical conductivity (2,222,222 S m^−1^), thermal conductivity (14 W m^−1^K^−1^) and density (7900 kg m^−3^) are for 304 stainless steel [37]. The metabolism was assumed to be 33,800 W m^−3^ [35] and blood flow was neglected because of the results presented by Edd *et al.* which show that perfusion stops during such a procedure [13]. For the experiments with immune-competent mice that had a delay between pulse trains (see Table 1), a 45 second delay was used. For the scenarios with immune-deficient mice, no delay between pulse trains was assumed.

### Histology, immunohistochemistry and DNA break detection

#### For haematoxylin–eosin-saffron (HES) staining

Tumors were fixed in Finefix (Milestone, Italy) and embedded in paraffin. Sections of 4 µm were prepared for routine HES staining.

#### For immunohistochemistry of microvessels CD31

Paraffin sections (4 µm-thick) were dewaxed and rehydrated. Endogenous peroxidase activity was quenched by 3% H_2_O_2_ for 10 min. Sections were placed in cover-plates (Shandon, Life Sciences Technology, Cergy-Pontoise, France) and incubated with blocking serum Power Block 1∶10 (BioGenex, San Ramon, CA, USA) for 10 min. The slides were then incubated for 1 h with purified rat-anti-mouse monoclonal anti-platelet endothelial cell adhesion molecule (PECAM-1 also called CD31), dilution 1∶300, (PharMingen, Heidelberg, Germany) followed by rabbit anti-rat immunoglobulins (Dako Denmark) dilution 1∶200, for 30 min, followed by PowerVision poly-HRP anti-Rabbit IgG (ImmunoVision Technologies, Brisbane, CA) for 20 min. Finally, slides were exposed to diaminobenzidine chromogenic substrate (DAB PowerVision Histostaining Kit; ImmunoVision Technologies) for 10 min, washed with distilled water, counterstained with Mayer's hematoxylin, and mounted in permanent medium (Pertex). All slides were immunolabelled the same day to ensure standardized intensities of immunochemical signals and counterstaining.

### TUNEL (Terminal deoxynucleotidyl transferase (TdT)-mediated dUTP Nick End-Labeling)

Double Strand DNA breaks, which are often associated with cell apoptosis, were detected using the “In Situ Cell Death” Detection kit (Roche; Mannheim, Germany) (TUNEL method) performed according to the manufacturer's instructions. De-paraffinized sections were incubated with Citrate buffer, pH 6 and placed in a water-bath, 98°C for 40 min and all sections were treated with TUNEL reagents (TUNEL mixture: 1 hour at 37°C under a coverslip) except for one where the enzyme was omitted (negative control). After washings with Rince Buffer Biogenex, slides were incubated with the secondary anti-fluorescein-AP conjugate, and the signal was revealed with Fast Red substrate solution for 20 min. Slides were lightly counterstained with hematoxylin prior to aqueous mounting by Aqua-Perm (Shandon Aqua-Perm™ Thermo Electron IVDD Compliant, Waltham, MA).

## Results

### Antitumour effects of irreversible electroporation

#### Effects on tumors transplanted in immunocompetent mice

**Figure 1:**
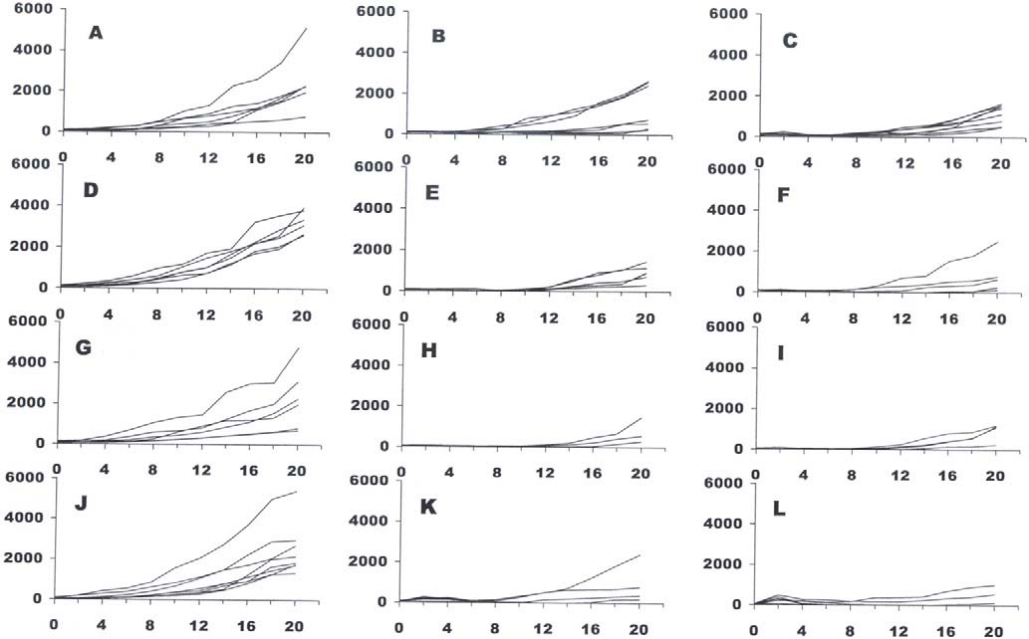
Determination of appropriate electrical parameters for an effective tumor treatment by IRE – experiments with immunocompetent mice. Panels are identified by letters corresponding to the parameters described in table 1, except for panel A (tumor growth in untreated mice followed as controls of treatment conditions B and C), panel D (control of E and F), panel G (control of H and I) and panel J (control of K and L).

Several electrical pulse parameters (Table 1) were tested to determine those providing a good tumour regression index. The initial parameters tested were derived from electrical parameters of reversible electroporation and employed an electric field (voltage to electrodes distance ratio) of 2000 V/cm and 800 µs total duration of the electric pulses (EP). These conditions did not result in any complete regression but, for some of the treated tumors, a slow-down of growth was recorded (Table 1 and Fig. 1B and 1C).

We tested additional modes of application of electrical pulses. In the following set of tests the EP were delivered in two perpendicular directions for a more complete coverage of the tumor. In some tests the number of EP was increased. These changes were only partly efficient as a slow down in tumour growth was again noticed but no complete regression (CR) was achieved, except for one single tumor (Table 1 and Fig. 1E and 1F). The major increase in efficacy on tumor regression resulted from the increase of the ratio of the applied voltage to electrodes distance. The voltage-to-distance ratio is used to characterize the “average” electric field strength in the tissue. The actual electric field in each point of the tissue depends on the geometry of the electrodes (not only the distance between the electrodes but also their shape and dimensions) and on the applied voltage. At 2500 V/cm, between 33% and 67% of complete regressions were achieved in the four experimental conditions tested (Table 1, Fig. 1H, 1I, 1K, and 1L). The efficacy does not seem to be related to the frequency, either 1Hz or 5000 Hz (Figs. 1I and 1K), at which the electric pulses were delivered.

#### Effects on tumors transplanted in immunodeficient mice

**Figure 2:**
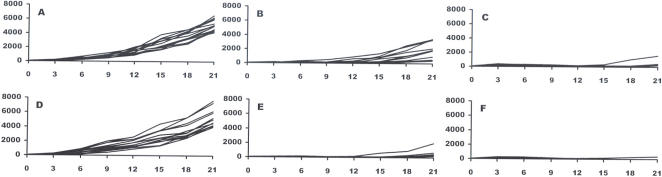
Determination of appropriate electrical parameters for an effective tumor treatment by IRE – experiments with immunodeficient (nude) mice. Panels are identified by letters corresponding to the parameters described in table 1, except for panel A (tumor growth in untreated mice followed as controls of treatment conditions B and C) and panel D (control of treatments E and F).

Similar results to those discussed above were achieved with tumors transplanted in nude mice to assess whether the host immune system has a low or moderate participation in the antitumor effects observed. A supplementary study confirmed that the immune response is not instrumental for IRE ablation which broadens the potential application space for IRE treatment to immunodepressed patients [38]. Another interesting point is that two different regimes were applied (80 EP of 100 µs versus 8 EP of 1000 µs; Table 1 and Fig. 2B and 2E, and 2C and 2F) which were characterized by the same EP total duration and by the same total treatment duration (26.7 s, 2B and 2C, or 267 s, 2E and 2F). The most effective electroporation parameters proved to be condition 2F that led to 12 CR out of 13 mice treated.

#### Analysis of thermal effects in the treated tumors

Table 1 contains two columns of results from the analysis of the thermal effects. One of the columns gives the maximum temperature associated with each procedure, and the second contains the duration required to maintain the tissue at 43°C in order to achieve an equivalent thermal dose as the procedure. The results are for the centerline, which is where the maximal temperature develops and can be considered as an upper limit. The analysis shows that the time delay between pulse trains allowed for significant heat dissipation through the electrodes. The results show that the heating was insignificant for cases 1B, 1C, 1E, 1F, 1I, 1K, 2C, 2E, 2F and most likely did not play a role during cases 1H, 1L and 2B. Since the model does not account for convective heat dissipation out of the sides and top of the tumor or of conduction though the animal itself, it is conservative and provides upper limits of the temperature increase in the tissue.

### Histological and immunohistochemical analysis

#### Evolution of the tumor cell morphology and tumor structure

**Figure 3:**
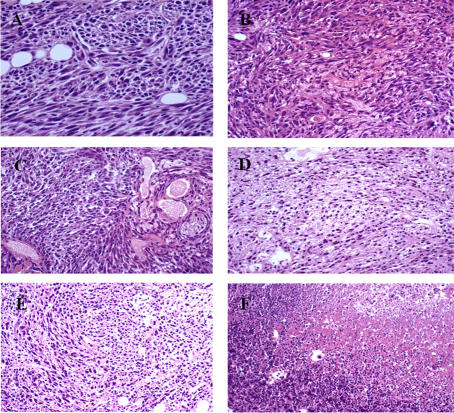
Analysis of tumor evolution by HES histological staining after IRE. A: control; B, C, D, E and F: respectively 1, 2, 6, 24 and 48 h after IRE.

In the untreated control tumors, cells display a large nucleus surrounded by a well marked cytoplasm and a well defined cell membrane (Fig 3A). The slides stained with the classical HES staining revealed that 5 min after the EP, no change in tumour cell morphology was detectable. However, the congestion of blood in the tumour vessels was evident. This effect is similar to the irreversible electroporation consequences in the liver described by Edd *et al.* [13]. Few changes in tissue architecture and cell morphology were detected 1 h and 2 h later (Fig 3B and 3C). However, 6 h after the EP, dramatic changes were observed (Fig 3D). Cytoplasmic limits between the cells were barely distinguishable in several parts of the tumour. The nuclei were still visible, but in a sort of syncytium in these parts of the tumour. At 24 h, almost the entire tumor presented this image (Fig 3F). The nuclei, extremely pycnotic, appeared to be all in the same “cytoplasm” as no limit was detectable between the original cells. At 48 h (Fig 3F) and 72 h, the nuclei were even smaller and tissue necrosis still more evident.

#### Analysis of cell death in the treated tumors

**Figure 4:**
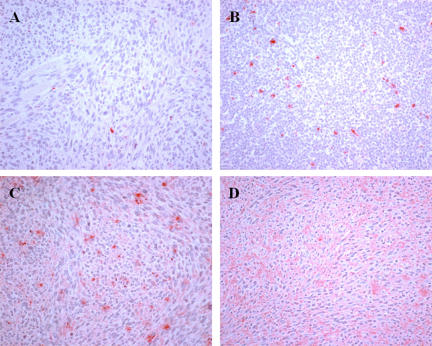
TUNEL analysis at different times after the pulses delivery. A: control; B, C and D: respectively 5 min and 1 and 24 h after IRE.

TUNEL staining revealed that in the control slides, very few cells were stained positive. The staining was strictly localized at the level of the cell nucleus (Fig 4A). Background was very clear (Fig 4A). However, as early as 5 min after the treatment, changes began, albeit slightly noticeable: a larger number of nuclei were positive, and in many of these nuclei, staining was not contained inside the nucleus, but was clearly spreading in the cytoplasm (Fig 4B). Background was still very clear (Fig 4B). The overall staining of the slices increased 1 h after EP delivery. There was a clear increase in the number of red-dye leaking cells and there was also the presence of a red background (Fig 4C). Thus, two types of staining can be clearly identified in these slices and at later times. At 6 h, a large number of cells still display the diffuse staining (not shown). However, at longer times (24 h, Fig 4D), there was only a heavily red-stained background, which completely diffused and continued to spread throughout the entire tumor section. At 24 h, no more red-dye leaking cells were found, in agreement with the HES images that showed the complete disintegration of the cells.

#### Evolution of the tumor vasculature

**Figure 5:**
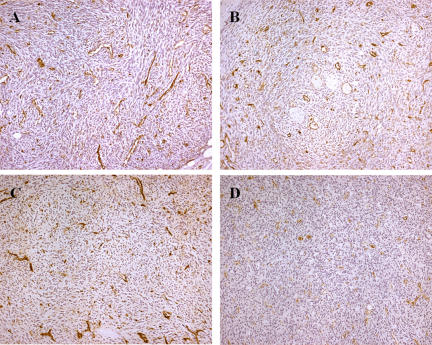
Immunohistochemical analysis of the tumour vasculature evolution by means of CD 31 staining in LPB tumors after treatment. A: control; B, C and D: respectively 2, 6 and 24 h after IRE.

Since congestion was detectable immediately after treatment in the HES slides, evolution of the tumour vasculature was also analysed using antibodies against the CD31 endothelial cells specific marker (Fig. 5). The changes in vasculature, which were observed 5 min after EP delivery were slight with respect to the controls (Fig. 5A), displayed highly branched and tortuous vasculature typical to this well vascularized fibrosarcoma tumor. However, 2 h later, well established vascular congestion with dilated vessels was detectable, long vessels were not visible and a slight diffuse staining began to spread in some parts of the slices. At 6 h, blood vessel walls, when still present, were either hyper- or hypo-pigmented. The diffusion of the CD31 marker in the extra-cellular interstitial spaces was much more intense and micro-vascular occlusions were also detectable. These signs indicate advanced tumour vasculature lesions. At 24 h, very advanced vascular lesions were seen, with faint CD31 marker staining of the endothelial cells indicating damaged blood vessel walls, and intense diffusion of the staining in the whole tissue. What remained were blood vessel skeletons over a necrotic background.

## Discussion

Our results provide evidence that IRE can be used to ablate tumors *in vivo*. The results achieved with the different sets of electrical parameters indicate that the main parameter affecting the results is the electric field strength. Trains of a large number of short pulses resulted in the best antitumor effects (up to 92% of tumor ablation).

From previous studies on the electric field distribution during electroporation *in vivo* [13], [39]–[41], the electrodes type (plates, different types of needle arrays) and the adequacy with which the IRE electrical field affects the totality of the tumor volume will be crucial parameters in achieving successful tumor ablation. In our study the electric pulses were delivered through parallel plates in direct contact with the tumor tissue, which is a configuration that ensures a rather homogeneous electrical field throughout the tumor [39]–[41]. Therefore, the values used in this study for the voltage to distance ratio are actually a good measure for the field that has developed in the treated tumors.

This study has produced several observations concerning the mechanisms of cell ablation in the IRE method as well as evidence of the efficacy of IRE to completely destroy aggressive tumors. As shown in normal liver tissue [13], classical HES staining revealed that the IRE pulses induce vascular congestion, which should also cause tissue hypoxia and may further contribute to tumor cell death. Twenty-four hours after the application of the pulses, all treated tissue was necrotic. Interestingly, the treatment does not produce massive apoptosis. The number of nuclei stained by the TUNEL reaction slightly increases 1 h after the treatment. The original and remarkable observation reported is the detection of diffused TUNEL staining first in the cytoplasm around the cell nucleus, and later, around the cells. In fact, this diffused staining, that constitutes almost the only TUNEL staining a few hours after the EP, is consistent with the expected effects of the applied IRE pulses. Indeed if irreversible electroporation is actually achieved, the plasma membrane should not reseal and there is also a possibility of affecting the nuclear envelope. Thus, DNA becomes accessible to extracellular nucleases (the DNA double strand breaks (DSB) are the seeding point for the TUNEL staining). Then DNA can spread out of the nucleus and out of the cell (facilitating the generation of DSB, the DSB themselves facilitating DNA spreading out of the nucleus). The observation of a diffused TUNEL staining corroborates that cells are no longer limited by their natural membrane barrier, which is indeed the hallmark of irreversible electroporation.

The analysis of the CD31 staining confirmed that membrane alteration due to IRE is the basis for the efficacy of the treatment. Initially, this staining was meant for the study of the evolution of the tumor vasculature disruption. Our results show rapid and severe lesions of the vasculature. They also reveal that the disaggregation of the membranes starts a couple of hours after the pulse delivery and becomes very intense 6 h later, and is complete at 24 h. The localization of the membrane antigen CD 31 at the cell membrane is progressive and becomes complete. We find that the antigen diffuses throughout the treated tumor volume, indicating the complete disruption of the membranes as well as cell necrosis.

The goal of our study was to evaluate the ability of the IRE electrical pulses to produce cell ablation through their effect on the cell membrane. However, electrical pulses can also have a thermal effect through electrical Joule heating. Since heating is also a well known mechanism for cell ablation, we designed our studies in such a way that the IRE electrical pulses we used produced the desired effect on the cell membrane without having a substantial thermal effect. Therefore, in treatment planning we designed for a mild and benign increase in temperature and used a very conservative mathematical model in the analysis, which does not account for convective heat dissipation through the air or conduction through the animal. With regards to the effects of temperature, it is interesting to compare the experiments 2B and 2C (Table 1 and Fig 2). According to our calculations, eight 1000 µs pulses produce more heating than eighty 100 µs pulses. A larger percentage of CR was achieved with the eighty 100 µs pulses than with the eight 1000 µs pulses. Thus, we can infer that heating cannot be the cause of tumor regression in IRE. Moreover, the higher tumor regression efficacy achieved by applying the same energy in a sequence of shorter pulses to induce less of a thermal dose is consistent with results achieved *in vitro* by Miller *et al.* [42]. In their study, 10 pulses of 0.3 ms (at 1500 V/cm) were more efficient in cell killing *in vitro* than 1 pulse of 3 ms (identical total duration) at the same voltage-to-distance ratio. Miller *et al.* also showed that complete cell killing was achieved using 3 sets of 10 pulses of 0.3 ms [42]. This results in a total pulse duration of 9 ms which is very close to the 8 ms used *in vivo* in our experiments.

The electrical pulses used in the experiments described in (2E and 2F) were chosen to yield an almost negligible thermal effect. The two experiments with 80 and 8 pulses yield an equivalent thermal dose of less than 1 s at 43°C. Even though it takes several tens of minutes of exposure at 43°C to retard the growth of murine fibrosarcoma [43], [44], condition 2F resulted in an impressive 92% of CR. Therefore our results, taken together, verify that the antitumor effects of IRE are not thermal. For an identical total energy delivery at the same electrical field, a large number of short pulses, which produce an overall lower tissue temperature, seem more effective than the use of a lower number of longer pulses. Furthermore, the use of long delays between pulses, which allows the tissue to cool, yields an overall lower tissue temperature thereby producing a very effective treatment.

In conclusion, our study has produced the first evidence that IRE can be used to effectively ablate tumors *in vivo*. Even though our results are encouraging, future studies can be conducted to optimize the results and explore the entire parameter space for this treatment. Nevertheless, the results achieved with different sets of electrical parameters suggest that the main parameters affecting the success of the treatment are not only the electric field strength, but also the number of pulses and the total pulse duration. The histological and immunohistochemical findings reported, as well as the theoretical arguments linked to the use of a high number of short pulses at a very low repetition frequency, demonstrate that tumor ablation is actually due to irreversible permeabilization of the tumor cells and not to excessive heating. It is worthy to note that TUNEL analysis of the cell death and CD31 analysis of the tumor vasculature evolution revealed not only the necrotic pathway of the IRE-caused cell death, but also confirms the mechanisms of the method, that is the actual irreversible electroporation of the cell membrane and its consecutive disintegration. This manuscript thus reports all the parameters necessary to bring IRE towards clinical trials that should help in defining the indications of this new cancer treatment.

## References

[1] Onik G, Rubinsky B, Zemel R, Weaver L, Diamond D (1991). Ultrasound-guided hepatic cryosurgery in the treatment of metastatic colon carcinoma. Preliminary results.. Cancer.

[2] Onik GM, Cohen JK, Reyes GD, Rubinsky B, Chang Z (1993). Transrectal ultrasound-guided percutaneous radical cryosurgical ablation of the prostate.. Cancer.

[3] Mouraviev V, Polascik TJ (2006). Update on cryotherapy for prostate cancer in 2006.. Curr Opin Urol.

[4] de Baere T, Rehim MA, Teriitheau C, Deschamps F, Lapeyre M (2006). Usefulness of guiding needles for radiofrequency ablative treatment of liver tumors.. Cardiovasc Intervent Radiol.

[5] Martin RC (2006). Hepatic tumor ablation: cryo versus radiofrequency, which is better?. Am Surg.

[6] Orlowski S, Mir LM (1993). Cell electropermeabilization: a new tool for biochemical and pharmacological studies.. Biochim Biophys Acta.

[7] Mir LM (2001). Therapeutic perspectives of in vivo cell electropermeabilization.. Bioelectrochemistry.

[8] Andre F, Mir LM (2004). DNA electrotransfer: its principles and an updated review of its therapeutic applications.. Gene Ther.

[9] Mir LM, Moller PH, Andre F, Gehl J (2005). Electric pulse-mediated gene delivery to various animal tissues.. Adv Genet.

[10] Huang Y, Rubinsky B (1999). Micro-Electroporation: Improving the efficiency and understanding of electrical permeabilization of cells.. Biomedical Microdevices.

[11] Davalos R, Huang Y, Rubinsky B (2000). Electroporation: Bio-electrochemical mass transfer at the nano scale.. Microscale Thermophysical Engineering.

[12] Davalos RV, Mir LM, Rubinsky B (2005). Tissue ablation with irreversible electroporation.. Ann Biomed Eng.

[13] Edd JF, Horowitz L, Davalos RV, Mir LM, Rubinsky B (2006). In vivo results of a new focal tissue ablation technique: irreversible electroporation.. IEEE Trans Biomed Eng.

[14] Rubinsky B, Onik G, Mikus P (2007). Irreversible electroporation: a new ablation modality–clinical implications.. Technol Cancer Res Treat.

[15] Mir LM, Orlowski S, Belehradek J, Paoletti C (1991). Electrochemotherapy potentiation of antitumour effect of bleomycin by local electric pulses.. Eur J Cancer.

[16] Mir LM, Glass LF, Sersa G, Teissie J, Domenge C (1998). Effective treatment of cutaneous and subcutaneous malignant tumours by electrochemotherapy.. Br J Cancer.

[17] Mir LM, Gehl J, Sersa G, Collins CG, Garbay JR (2006). Standard Operating Procedures of the Electrochemotherapy.. Eur J Cancer Supplements.

[18] Marty M, Sersa G, Garbay JR, Gehl J, Collins CG (2006). Electrochemotherapy - an easy, highly effective and safe treatment of cutaneous and subcutaneous metastases: results of the ESOPE (European Standard Operating Procedures of Electrochemotherapy) study.. Eur J Cancer Supplements.

[19] Deng J, Schoenbach KH, Buescher ES, Hair PS, Fox PM (2003). The effects of intense submicrosecond electrical pulses on cells.. Biophys J.

[20] Beebe SJ, White J, Blackmore PF, Deng Y, Somers K (2003). Diverse effects of nanosecond pulsed electric fields on cells and tissues.. DNA Cell Biol.

[21] Gowrishankar TR, Weaver JC (2006). Electrical behavior and pore accumulation in a multicellular model for conventional and supra-electroporation.. Biochem Biophys Res Commun.

[22] Belehradek M, Domenge C, Luboinski B, Orlowski S, Belehradek J (1993). Electrochemotherapy, a new antitumor treatment. First clinical phase I–II trial.. Cancer.

[23] Gothelf A, Mir LM, Gehl J (2003). Electrochemotherapy: results of cancer treatment using enhanced delivery of bleomycin by electroporation.. Cancer Treat Rev.

[24] Sersa G, Cemazar M, Rudolf Z (2003). Electrochemotherapy: advantages and drawbacks in treatment of cancer patients.. Cancer Therapy.

[25] Sersa G (2006). The State-of-the-art of electrochemotherapy before the ESOPE study; advantages and clinical uses.. Eur J Cancer Supplements.

[26] Nuccitelli R, Pliquett U, Chen X, Ford W, James Swanson R (2006). Nanosecond pulsed electric fields cause melanomas to self-destruct.. Biochem Biophys Res Commun.

[27] Belehradek J, Barski G, Thonier M (1972). Evolution of cell-mediated antitumor immunity in mice bearing a syngeneic chemically induced tumor. Influence of tumor growth, surgical removal and treatment with irradiated tumor cells.. Int J Cancer.

[28] (1998). United Kingdom Co-ordinating Committee on Cancer Research (UKCCCR) Guidelines for the Welfare of Animals in Experimental Neoplasia (Second Edition).. Br J Cancer.

[29] Becker SM, Kuznetsoz AV (2006). Numerical Modeling of In Vivo Plate Electroporation Thermal Dose Assessment.. ASME J of Biomechanical Engineering.

[30] Damianou CA, Hynynen K, Fan X (1995). Evaluation of accuracy of a theoretical model for predicting the necrosed tissue volume during focused ultrasound surgery.. IEEE Transactions on Ultrasonics, Ferroelectrics and Frequency Control.

[31] Sapareto S, Dewey W (1984). Thermal dose determination in cancer therapy.. Int J radiation oncology Biol Phys.

[32] Davalos RV, Rubinsky B, Mir LM (2003). Theoretical analysis of the thermal effects during in vivo tissue electroporation.. Bioelectrochemistry.

[33] Incropera FP, DeWitt DP, Incropera FP, DeWitt DP (2002). Chapter 5: Transient Conduction.. Introduction to Heat Transfer.

[34] White FM (1988). Chapter 1: Introduction. Heat and Mass Transfer.

[35] Deng ZS, Liu J (2001). Blood perfusion-based model for characterizing the temperature fluctuations in living tissue.. Phys A STAT Mech Appl.

[36] Swarup A, Stuchly SS, Surowiec A (1991). Dielectric properties of mouse MCA1 fibrosarcoma at different stages of development.. Bioelectromagnetics.

[37] White FM (1988). Appendix C: Properties of Metallic Solids Heat and Mass Transfer.

[38] Al-Sakere B, Bernat C, André F, Connault E, Opolon P, Davalos RV, Mir LM (2007). A study of the immunological response to tumor ablation with irreversible electroporation.. Technol Cancer Res Treat.

[39] Miklavcic D, Corovic S, Pucihar G, Pavselj N (2006). Importance of tumour coverage by sufficiently high local electric field for effective electrochemotherapy.. Eur J Cancer Supplements.

[40] Miklavcic D, Beravs K, Semrov D, Cemazar M, Demsar F (1998). The importance of electric field distribution for effective in vivo electroporation of tissues.. Biophys J.

[41] Miklavcic D, Semrov D, Mekid H, Mir LM (2000). A validated model of in vivo electric field distribution in tissues for electrochemotherapy and for DNA electrotransfer for gene therapy.. Biochim Biophys Acta.

[42] Miller L, Leor J, Rubinsky B (2005). Cancer cells ablation with irreversible electroporation.. Technol Cancer Res Treat.

[43] Hahn EW, Alfieri AA, Kim JH (1978). Single dose X-irradiation and concomitant hyperthermia on a murine fibrosarcoma.. Cancer.

[44] Mohamed F, Stuart OA, Glehen O, Urano M, Sugarbaker PH (2004). Docetaxel and hyperthermia: factors that modify thermal enhancement.. J Surg Oncol.

